# Off-Grid DOA Estimation Based on Circularly Fully Convolutional Networks (CFCN) Using Space-Frequency Pseudo-Spectrum

**DOI:** 10.3390/s21082767

**Published:** 2021-04-14

**Authors:** Wenqiong Zhang, Yiwei Huang, Jianfei Tong, Ming Bao, Xiaodong Li

**Affiliations:** 1 Key Laboratory of Noise and Vibration Research, Institute of Acoustics, Chinese Academy of Sciences, Beijing 100190, China; zhangwenqiong@mail.ioa.ac.cn (W.Z.); huangyiwei@mail.ioa.ac.cn (Y.H.); tongjianfei@mail.ioa.ac.cn (J.T.); lxd@mail.ioa.ac.cn (X.L.); 2 University of Chinese Academy of Sciences, Beijing 100049, China

**Keywords:** off-grid, DOA estimation, circularly fully convolutional networks, space-frequency pseudo-spectrum, high resolution

## Abstract

Low-frequency multi-source direction-of-arrival (DOA) estimation has been challenging for micro-aperture arrays. Deep learning (DL)-based models have been introduced to this problem. Generally, existing DL-based methods formulate DOA estimation as a multi-label multi-classification problem. However, the accuracy of these methods is limited by the number of grids, and the performance is overly dependent on the training data set. In this paper, we propose an off-grid DL-based DOA estimation. The backbone is based on circularly fully convolutional networks (CFCN), trained by the data set labeled by space-frequency pseudo-spectra, and provides on-grid DOA proposals. Then, the regressor is developed to estimate the precise DOAs according to corresponding proposals and features. In this framework, spatial phase features are extracted by the circular convolution calculation. The improvement in spatial resolution is converted to increasing the dimensionality of features by rotating convolutional networks. This model ensures that the DOA estimations at different sub-bands have the same interpretation ability and effectively reduce network model parameters. The simulation and semi-anechoic chamber experiment results show that CFCN-based DOA is superior to existing methods in terms of generalization ability, resolution, and accuracy.

## 1. Introduction

Direction of arrival (DOA) estimation is an important research direction in array signal processing, and it has been widely used in many military and civilian fields such as radar, communications, sonar, seismic, exploration and radio astronomy [1,2]. In many application scenarios, such as the Internet of Things and unattended ground sensor (UGS) systems [3], which focus on the remote targets, e.g., vehicles or helicopters, detection in the field, the array aperture is strictly limited so that it far exceeds the Rayleigh limit, which has made low-frequency multi-source DOA estimation complex problem for a long time.

Among traditional DOA estimation algorithms, subspace-based methods are considered to be high-resolution, such as MUSIC (multiple signal classification) [4] and ESPRIT (estimation of signal parameters via rotational invariance techniques) [5]. MUSIC-based methods employ the orthogonality of the signal subspace (steering vectors) and the noise subspace to search the spatial spectrum to achieve high-resolution. ESPRIT-based methods avoid spectrum search by the signal subspace rotation invariance properties and reduce computational complexity. When the uncertainty of the system or background noise leads to model errors, e.g., the wrong number of sources, subspace-based methods need to solve high-dimensional non-linear parameter estimation problems. Although many improved algorithms [6,7,8,9] based on MUSIC and ESPRIT have been developed to estimate the number of sources jointly, sometimes in order to solve the singular matrix of the spatial covariance, they may sacrifice the array aperture [10] and deteriorate the resolution. Later, compressed sensing (CS)-based methods [11,12,13] have been widely studied due to the consideration of both the super-resolution capability and the ability to detect the number of sources by exploiting spatial spectrum sparsity. Nevertheless, they suffer from a large amount of computational load caused by on-grid or off-grid search, especially in the case of wideband, which is challenging to apply to engineering implementation.

Alternatively, machine learning-based approaches, e.g., an artificial neural network (ANN), can provide a means of mapping from input features to DOA [14,15,16,17,18]. Moreover, ANNs consist of elementary mathematical calculations and have an advantage in computing speed compared with conventional DOA estimation algorithms. At the earliest, the radial basis function (RBF) neural network [19] is introduced to DOA estimation, which successfully learns the single source direction finding function from data sets despite the lack of resolution. Then, ref. [20] employs support vector regression (SVR) to improve the DOA resolution, while SVR is a small sample set learning method with a solid theoretical foundation and limits its application in practice. To this end, ref. [14] utilizes a multilayer perceptron (MLP) neural network to enhance the non-linear interpretation ability to the DOA mapping model. However, as the number of MLP network layers increases, the generalization performance may not necessarily be improved, which means that the accuracy of DOA estimation is insufficient.

In terms of generalization performance, deep learning has made significant progress, and the generalization performance is further improved as the training set increases. Various methods for DOA estimation based on deep learning have been proposed [21,22,23,24,25,26,27]. In [21], a deep neural network (DNN) was devised to perform logistic regression for each DOA to achieve high accuracy, while this method requires the known number of sources. To overcome this drawback, refs. [24,26] formulate multi-source DOA estimation as a multi-label multi-classification problem by convolutional neural networks (CNNs) and convolutional-recurrent neural networks (CRNNs), respectively, while the accuracy depends on the number of grids. Ref. [27] employs DNN to achieve rough candidate DOAs, and then takes the method of amplitude interpolation to estimate the signal directions of non-integer impinging angles. Similarly, ref. [25] first adopted CRNNs as spatial filters to obtain rough candidate sectors and then used classifiers to extract the precise directions. In [23], MLP neural networks are adopted to estimate possible sub-areas, and RBF neural networks are utilized for fine position estimation. Ref. [22] uses the multitask autoencoder for spatial filtering and then realizes spatial spectrum estimation by a fully connected multilayer neural network. From DP-based object detection models such as Faster R-CNN [28], and RefineDet++ [29] in the image field, we can learn that high-accuracy object position can be obtained from region proposal networks and regression refinement networks. Based on this inspiration, ref. [30] developes a two-stage cascaded neural network for DOA estimation, which includes a CNN and a DNN-based regressor for the discrete angular grid and the mismatch between true DOAs and discrete grids. However, the CNN-based on-grid method mainly focuses on the narrowband signal case. The input of the regressor, including the input and output of the first-stage CNN, may increase the complexity of the training data set. As can be seen from the above, high-resolution multi-source DOA estimation usually has two-stage networks for coarse search and fine search. Note that the DOA classifiers used by these algorithms map the features of all frequency sub-bands to the spatial spectrum. This means that the generalization ability of the model is sensitive to the frequency characteristics of the training data. In this way, the training data set samples are usually required to be large enough, making it difficult to exhaust all types of sources in practical applications.

This paper proposes a new off-grid DOA estimation method, which has two networks: (i) An on-grid multi-label classifier based on circularly fully convolutional networks (CFCNs) which was devised for rough grid proposals. The classifier provides the mapping from the phase map to the space-frequency pseudo-spectrum. Moreover, by performing circular convolution calculation on the sensing axis, the phase features at each sub-band can be extracted to a greater extent than linear convolution. To achieve high-resolution DOA grid proposals, the CFCN increases the feature dimension in exchange for an increase in space dimension by rotating convolutional networks; (ii) Based on these grid proposals, an off-grid regression network which was developed for precise DOA adjustment. The regressor obtains the actual deviation in the grid-gap from the features produced by the CFCN. The proposed models are trained by synthetic single-source white noise signals, which avoids the tedious and exhaustive data set. The main contributions of the proposed method are as follows:

- The circular convolution calculation enhances the phase feature acquisition ability.
- The CFCN ensures that the DOA estimations at different sub-bands have the same interpretation ability and effectively reduces network model parameters.
- The proposal DOA grids and the corresponding features provide a more feasible traversal within the grid-gap for the regression training data set and reduce the complexity of the data set.

The simulation and semi-anechoic chamber experiment results show that under the conditions of single/dual-source targets with different band-limited/signal-to-noise ratios (SNR)s, the CFCN-DOA is superior to existing methods in terms of generalization ability, resolution, and accuracy.

The rest of this paper is organized as follows: Section 2 introduces the problems of deep learning models on DOA estimation; Section 3 describes details of the off-grid DOA estimation model based on CFCNs, data set generation methods, and trained model performance comparison results with different parameters; Section 4 carries out simulation results and performance evaluation; Section 5 shows experimental verification; Section 6 discusses the experiments; and Section 7 summarized the whole work.

## 2. Problem Formulation

For the off-grid multi-source DOA estimation problem, we first need to determine the inputs and outputs of the model. The problem is split into two sub-problems, i.e., on-grid and off-grid problems, which are formulated as a multi-label and multi-classification problem and a regression problem, respectively. The multi-label classifier provides the DOA proposals, and the regressor produces precise DOA in the corresponding grid-gap, which fully meets the requirement of precise multi-source orientation.

In a model based on deep learning, the input is required to have sufficient information representation. For micro-aperture arrays detecting far-field sources, the signal strength features received by different sensors are not significant. Thus, DOA mainly depends on the phase difference between different sensors at different frequencies. In this paper, the phase map is chosen as the input of the model, and it is a M×F matrix $\Phi_{k}$ at time *k*, where *M* is the number of sensors and *F* is the number of all sub-band signals. $\Phi_{k}$ is denoted as

(1) $$\begin{array}{c} \Phi_{k}=\left[\begin{array}{ccc} \phi_{k,1,1} & \cdots & \phi_{k,1,F}\\ \vdots & \ddots & \vdots \\ \phi_{k,M,1} & \cdots & \phi_{k,M,F} \end{array}\right], \end{array}$$

where $\phi_{k,m,f}$ is the phase of the received signal of the *m*-th sensor at the *f*-th sub-band, which is obtained by a *N* point discrete Fourier transform (DFT).

**Figure 1:**
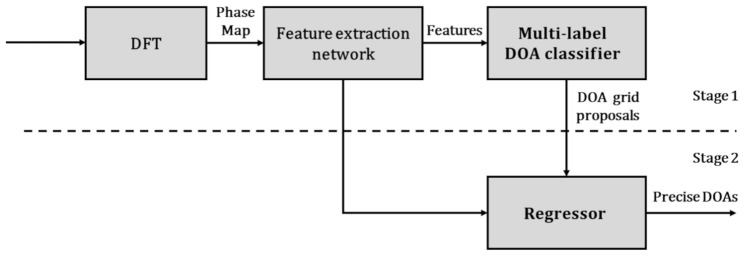
Block diagram of the proposed off-grid direction of arrival (DOA) estimation.

The outputs of the model should be accurate DOAs of multiple sources, while it is challenging to obtain DOAs directly when the number of sources is unknown. Therefore, we formulate the model as a two-stage network, which is shown in Figure 1. At stage 1, this is an on-grid problem. Firstly, all the raw signals are transformed into the phase map by DFT. Then, all the features are extracted by the feature extraction network. Finally, the multi-label classifier provides DOA grid proposals. At stage 2, based on the features and DOA grid proposals given by the stage 1, the regressor produces precise DOAs. The network at stage 1 is first trained with the data set labeled by DOA grids, and then the regressor is trained with the data set labeled by precise DOAs in the grid-gap.

In this paper, we assume that there is only one source in a grid. The problem of the multi-label classifier at stage 1 is to obtain reliable DOA grid proposals based on the phase features of all sub-bands by using the space-frequency pseudo-spectrum. In addition, the problem of the regressor at stage 2 is to choose sufficient features as its inputs.

## 3. Off-Grid DOA Estimation

This section focuses on the proposed architecture, the generation of the data set, and the design of structural parameters in the network.

### 3.1. The Off-Grid DOA Estimation Based on Circularly Fully Convolutional Networks (CFCNs)

**Figure 2:**
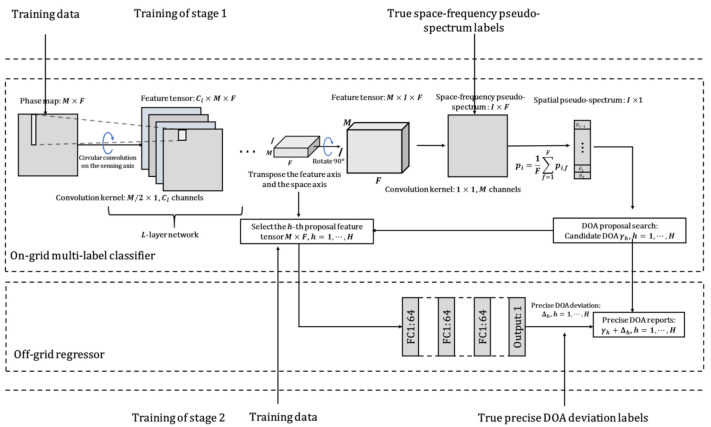
Proposed architecture.

In the CNN convolution calculation, the linear convolution calculation used may ignore some sensor phase difference features, which are probably important information, caused by the most spaced sensors. If this phase difference is ignored, it may affect the target resolution performance. To learn the phase features of each sub-band to the greatest extent, the model needs to have the same interpretation capability for each sub-band. We develop a circularly fully convolutional network (CFCN)-based architecture consisting of two networks: the on-grid multi-label classifier and the off-grid regressor. This architecture is shown as Figure 2.

For the multi-label classifier, to ensure the independence between the spectral features of the source and space, the size of the convolution kernel is designed to be M/2×1. When performing circular convolution calculation, the input phase matrix needs to be extended by M/2−1 length in the spatial dimension, i.e., the sensing axis. The feature extraction network has *L* layers, and each layer has $C_{l}$ neurons/channels, where $C_{l}$ can be called the feature dimension. The neurons are activated by rectified linear unit (ReLU). Since the output dimension of the fully convolutional network (FCN) is consistent with the input dimension, up-sampling is usually used to achieve dimension upgrades in a certain dimension. Up-sampling does not increase the total amount of features/information but increases data storage. In this paper, we can increase the feature dimension in exchange for increasing the space dimension by rotating the convolutional network, i.e., transposing the feature dimension and the space dimension. In the last layer of the convolution feature extraction layers, the number of neurons is consistent with DOA grids. Finally, the 1×1 convolution layer fuses the phase features of all sensors at each sub-band. To obtain the posterior probability $p(\theta_{i,f}|\Phi_{k})$ of DOA at the *i*-th space grid and the *f*-th sub-band at time *k*, the last layer is activated by the Sigmoid function, where $\theta_{i,f}$ represents the direction corresponding to the *i*-th DOA class at the *f*-th sub-band. The I×F posterior probability matrix is the space-frequency pseudo-spectrum, where *I* is the number of space grids and *F* is the number of sub-bands. For the multi-source DOA estimation, the pseudo-posterior probability of each DOA class is obtained by averaging the probabilities of all sub-bands:

(2) $$\begin{array}{c} p\left(\theta_{i}|\Phi_{k}\right)=\frac{1}{F}\sum_{f=1}^{F}p\left(\theta_{i,f}|\Phi_{k}\right). \end{array}$$

From the pseudo-posterior probabilities, *H* DOA classes corresponding to the *H* peaks with the highest probability are selected as candidate DOAs $\gamma_{h}$, h=1,⋯,H, which are treated as DOA proposals for the regressor. In this work, we use a simple peak detection method as the DOA proposal search to verify the effectiveness of the proposed algorithm.

Before the last 1×1 convolution layer fusing features of all sensors, the M×I×F feature tensor *R* has the sufficiency of the DOA information in the grid-gap. On-grid methods cannot provide precise DOA estimation. Thus, the regression-based off-grid method is proposed. For this regressor, the accurate DOA deviation $\Delta_{h}$ of the DOA proposal $\gamma_{h}$ is mapped from the *h*-th proposal feature M×F matrix. Therefore, the final off-grid DOA $\varphi_{h}$ is:

(3) $$\begin{array}{c} \varphi_{h}=\gamma_{h}+\Delta_{h},h=1,\cdots ,H. \end{array}$$

In the training process, the on-grid multi-label classifier is trained first, and then the on-grid regressor is trained.

### 3.2. The Generation of the Data Set

For the case of long-distance, low-frequency, and low-SNRs, this article does not temporarily consider the influence of complex reverberation/multi-path. Under different targets, different source operating states, and single-source/multi-source, the signals received by the array are different. If the signals with different features at different locations are exhaustively enumerated under different SNR conditions, the data set will be extensive, and it cannot cover all combinations. In addition, when the signal frequency ranges of different targets overlap, the non-linear superposition of different sources will make the entire network challenging to train.

**Figure 3:**
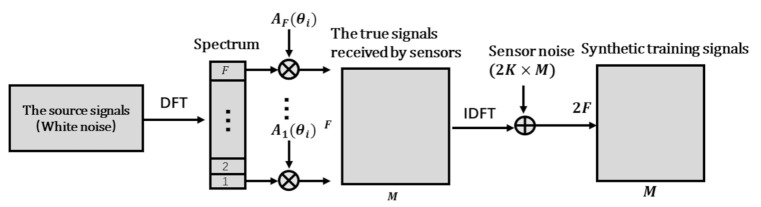
The generation process of data set signals.

For the data set of the multi-label classifier, signals received by the array are generated by a single white noise source traversing different orientations and different SNRs. In this way, the features of all sub-bands vs. space orientations can be represented. The generation process is shown in Figure 3. First, the single-source white noise is transformed to the frequency domain by DFT, and the phase compensation is performed for each sub-band signal to obtain the true signal received by each sensor, where the compensation factor is the steering vector $A_{f}\left(\theta_{i}\right)$, f=1,⋯,F. The corresponding label is a I×F space-frequency pseudo-spectrum matrix, whose *i*-th row is equal to 1, and the other items are 0. Then, the true signals are converted into the time domain by inverse discrete Fourier transform (IDFT), mixing the sensor noise. Considering the worse noise conditions, the SNR is subject to [−6dB,60dB] uniform random distribution. The training data set referred to in this article has I×4000 samples, and the testing set has I×400 samples.

For the data set of the regressor, the inputs are the features extracted by the multi-label classifier from the phase map, and the labels are the accurate DOA deviations in the grid-gap. If this array is symmetrical, the mapping between the DOA deviation and the feature is consistent for each grid-gap uniformly divided in space. Therefore, we can train the regressor in one grid-gap to generalize to other grid-gaps. The signals of the training set can still be traced to the original phase map input, which is generated by the process in Figure 3 according to the new DOA parameter $\varphi_{i}$

(4) $$\begin{array}{c} \varphi_{i}=\theta_{i}+\Delta_{i}, \end{array}$$

where $\Delta_{i}$ is the DOA deviation at the *i*-th grid-gap, and it is also the label of the *i*-th proposal feature M×F matrix. $\Delta_{i}$ is subject to [−g/2,g/2] uniform random distribution, where *g* is the DOA width of a grid-gap. Other parameter settings refer to parameters of the data set in the multi-label classifier. The numbers of samples in training and testing sets are 4000 and 400, respectively.

### 3.3. Training Methods and Results

The proposed deep networks are realized and trained in Pytorch on a PC with a single graphic processing unit (GPU) RTX2080Ti and an Intel i7-8700 processor. We use the stochastic gradient descent algorithm with a momentum of 0.9 to train the CFCN-based multi-label classifier and the regressor. The number of samples in each batch is set as *I*, binary cross-entropy is used as the loss function for the multi-label classifier, and mean-squared error (MSE) is used for the regressor. The cyclic learning rate scheduler [31] is used for training, and the learning rate range is from $10^{-6}$ to $10^{-1}$. Xavier [32] is used for initialization. Note that this model only uses a single-source white noise data set as training, and no other multi-source band-limited signal data sets are used for training here.

In this work, we take a uniform circular array with parameters of M=8.70 mm aperture as an example to compare the performance of different network structures. The number of DOA grids is I=72, i.e., the width of the grid-gap is $5^{\circ }$. The sampling frequency is Fs = 3 kHz, the length of each snapshot is N=512, and then the number of sub-bands is F=N/2=256, up to the Nyquist frequency. The influence of different convolution kernel sizes, different convolution layer widths, and different convolution layer numbers on the parameter quantity and performance of the model is discussed. The evaluation results are shown in Table 1. The structure of the model in Figure 2 is encoded for convenience. “L*” means the number of convolutional network layers is “*”. “FC” means a fully connected network. “K*” means the size of the convolution kernel ∗×1. “[·]×∗” means “·” repeated “*” times. For example, CNN + FC: L7-K2- [64] × 7 + FC[[512] × 2, 72] means: the model consists of seven convolutional layers, the convolution kernel size is 2 × 1, each layer has 64 channels, and the fully connected network structure is [512 × 512 × 72].

Table 1 shows the comparison of different model parameters, accuracy, and total floating point operations (FLOPs), which are obtained using an open source neural network analyzer (https://github.com/Swall0w/torchstat (accessed on 31 March 2021)). We can see that although CFCN-based methods slightly increase computational complexity, they have fewer parameters and higher accuracy than CNN + FC [24]. The main reason is that a fully connected network occupies a lot of parameters, and the phase difference features between sensors will decrease significantly as the frequency decreases. All sub-band features extracted by the CNN are input to the fully connected network, and then the fully connected network may sacrifice low phase difference features in the low-frequency range to highlight the important feature contributions in the high-frequency range in order to improve the score in training. This leads to a weak generalization ability on low-frequency signals. This inference can be verified in the simulation experiment in the next section.

The CFCN does not use a fully connected network to classify all features but uses a separate phase feature classification for each sub-band. In this way, it effectively reduces the parameters and ensures that each sub-band feature has an equal contribution to the DOA estimation.

When the CFCN has a 4×1 convolution kernel, the DOA estimation accuracy rate increases rapidly as the number of convolutional network layers increases. When the convolutional layer width is 72 channels, and the number of convolutional layers is increased to six layers, the accuracy rate reaches the highest 98.5%. When the width of the convolutional layer is 128, the number of convolutional layers can be up to five, and the accuracy rate is 95.1%. If the size of the convolution kernel is reduced to 2×1, the convolution width is set to 72, the number of convolution layers can be up to 11, and the accuracy rate can reach 97.8%. Therefore, a fully convolutional network with an *I* width of M/2+2 layers is used in the following text.

For the regressor, we utilized a four-layer fully connected network to approximate the mapping between the deviation of DOA in the grid-gap and the corresponding features. After training, the final mean absolute error (MAE) of the off-grid DOA reaches $0.782^{\circ }$.

### 3.4. Computational Complexity

In the implementation of computational processing, deep learning-based methods have been more optimized for parallel computing than traditional subspace-based algorithms such as MUSIC. Table 2 shows the average computing time of different methods on the platform of central processing unit (CPU) and GPU used by this paper. Deep learning-based DOA algorithms have better real-time performance. For the wide-band MUSIC, the total computational complexity is $O(FM^{3}+FM^{2}I)$ [33], where $O(FM^{3})$ and $O(FM^{2}I)$ are the complexity of eigen-decomposition and the spatial pseudo-spectrum search for *F* sub-bands, respectively. According to [34] and network structures involved in the article, the computational complexity of the CFCN and CNN-DOA are $O(\frac{M^{3}}{4}I^{2}F)$ and O(M×MF×M/2×64×64+2×64×F×512+2×512×I), respectively. As the numbers of sensors and DOA grids increase, the proposed method has more computational complexity than CNN-DOA. If the input signals overlap 50%, i.e., the report refresh period is 256/3000=0.085s, the CFCN based on the structure L6-K4-[72] × 6 requires a processing speed of more than 2.52 GigaFLOPs (GFLOPs) per second (FLOPs/S). For the current embedded processors such as the RK3399Pro IoT device with the neural process engine reaching 2.4 TeraFLOPs/S (TFLOPs/S) [35] and NVIDIA TX2 with compute unified device architecture (CUDA) cores reaching 1.5 TFLOPs/S [36], the calculation requirements of CFCN-based methods are quite acceptable. The CFCN can even be implemented in a full-hardware system [37] for better real-time performance.

## 4. Simulation Experimental Evaluation

In this section, simulation experiments are implemented to evaluate the generalization ability and DOA estimation performance of the trained network under different conditions.

### 4.1. Baselines and Objective Measures

The performance of the proposed method is compared to two common algorithms: MUSIC [4] and CNN-based DOA [24]. To ensure a fair comparison, we set similar parameter settings for other methods, e.g., the DOA grid is $5^{\circ }$. The wideband MUSIC method averages the spatial pseudo-spectrum of all sub-bands to obtain the wideband spatial pseudo-spectrum. The *H* highest peak values are selected as the final DOA estimates. Two hundred Monte Carlo experiments were performed for the statistics.

For the objective evaluation, OSPA [38] (optimal sub-pattern assignment) was used as the multi-source DOA error metric:

(5) $$\left\{\begin{array}{cc} D_{p,c}\left(X,Y\right)={\left[\frac{1}{\left|X\right|}\left(\min_{\pi \in \Pi_{\left|X\right|}}\sum_{i=1}^{\left|X\right|}d_{c}^{p}\left(x_{i},y_{i}\right)+\left(|X\right|-\left|Y|\right)\cdot c^{p}\right)\right]}^{\frac{1}{p}},\; & \left|X|\leq |Y\right|\\ D_{p,c}\left(X,Y\right)={\left[\frac{1}{\left|X\right|}\min_{\pi \in \Pi_{\left|X\right|}}\sum_{i=1}^{\left|X\right|}d_{c}^{p}\left(x_{i},y_{i}\right)\right]}^{\frac{1}{p}},\; & \left|X|>|Y\right| \end{array},\right.$$

where X is the measured DOA set, Y is the true DOA set, $x_{i}\in X$, $y_{i}\in Y$, |·| is the cardinality of the set ·, *p* is the order of OSPA, $\Pi_{\left|X\right|}$ is the set of |X| elements extracted and permuted and combined from Y, and $d_{c}\left(x,y\right)$ is the cut-off distance:

(6) $$\begin{array}{c} d_{c}\left(x,y\right)=\min\left\{c,d\left(x,y\right)\right\}. \end{array}$$

In (6), d(x,y) is the difference between two angles:

(7) $$\begin{array}{c} d\left(x,y\right)=\min\{|x-y|,|360^{\circ }-|x-y||\}. \end{array}$$

In this article, we set that $c=45^{\circ }$, and p=2. For the *J* Monte Carlo tests, the mean OSPA ${\bar{D}}_{p,c}$ is:

(8) $$\begin{array}{c} {\bar{D}}_{p,c}=\frac{1}{J}\sum_{j=1}^{J}D_{p,c}\left(x_{i},y_{i}\right). \end{array}$$

For the single-source case, the mean OSPA is the MAE. If the mean OSPA is more than $20^{\circ }$, the algorithm is evaluated as a failure.

### 4.2. Simulation Experiments

#### 4.2.1. Simulation Settings

The sampling frequency of the input signal is Fs = 3 kHz, and the data length of each time frame is N=512. To evaluate the performance of the model, we design challenging simulation conditions including different bandwidth-limited signal sources, single and dual sources, and different SNRs, where the dual sources are angularly separated by $135^{\circ }$, and the SNR range is [−6dB,0dB,6dB,12dB,20dB]. The specific simulation conditions are as follows:

(1) Single-source situation:

- Low frequency: 0–200 Hz;
- Full frequency: 0–1500 Hz;

(2) Dual-source situation:

- Overlapping low frequency: 0–200 Hz;
- Non-overlapping frequency: 0–200 Hz, 200–500 Hz;
- Overlapping full frequency: 0–1500 Hz;

#### 4.2.2. Simulation Results

**Figure 4:**
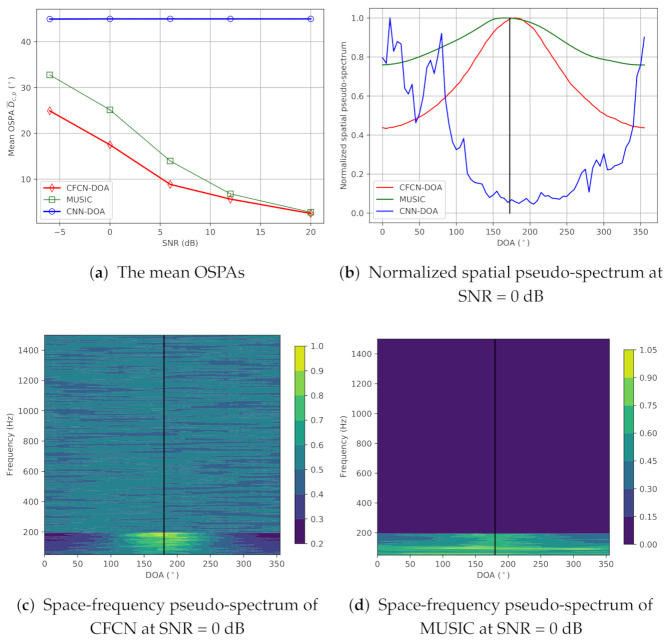
The results of the simulation under the low-frequency single-source condition.

**Figure 5:**
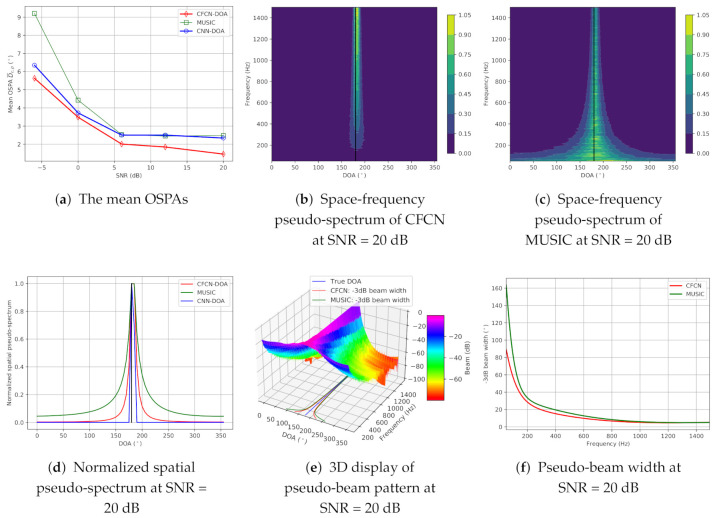
The results of the simulation under the full-frequency single-source condition.

**Figure 6:**
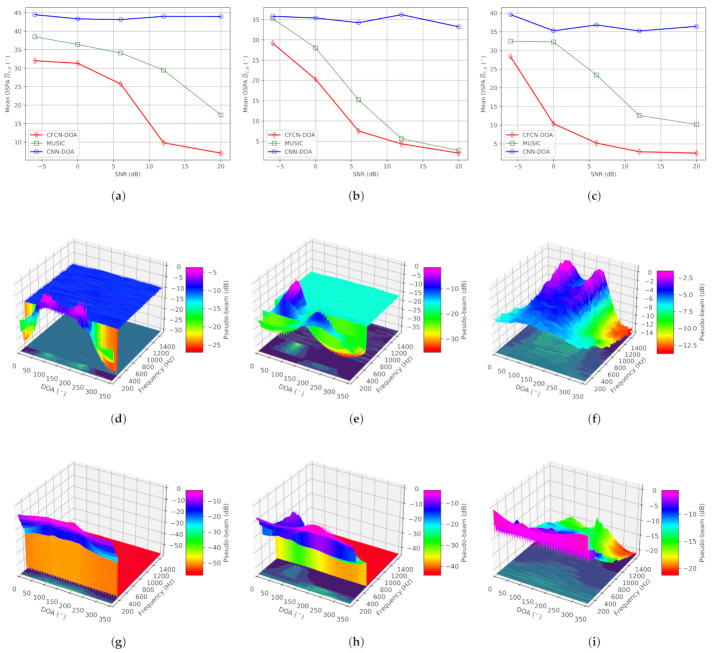
The results of the simulation under the dual-source condition. (**a**) The mean OSPAs under the overlapping low-frequency condition. (**b**) The mean OSPAs under the non-overlapping frequency condition. (**c**) The mean OSPAs under the overlapping full-frequency condition. (**d**) Space-frequency pseudo-spectrum of CFCN under the conditions: overlapping low-frequency and SNR = 12 dB. (**e**) Space-frequency pseudo-spectrum of CFCN under the conditions: non-overlapping frequency and SNR = 6 dB. (**f**) Space-frequency pseudo-spectrum of CFCN under the conditions: overlapping full-frequency and SNR = 0 dB. (**g**) Space-frequency pseudo-spectrum of MUSIC under the conditions: overlapping low-frequency and SNR = 12 dB. (**h**) Space-frequency pseudo-spectrum of MUSIC under the conditions: non-overlapping frequency and SNR = 6 dB. (**i**) Space-frequency pseudo-spectrum of MUSIC under the conditions: overlapping full-frequency and SNR = 0 dB.

According to the above simulation test conditions, the simulation results of CFCN-DOA, MUSIC and CNN-DOA for the 200 Monte Carlo tests are as follows:

(i) Single-source situation:

(a) Low-frequency band-limited source cases: The true DOA is set to $182.5^{\circ }$ in the middle of grids. Then, the on-grid-based methods cannot provide the precise DOA. Simulation evaluation results for the low-frequency case are shown in Figure 4. Figure 4a shows the mean OSPAs (MAEs) of three methods over different SNRs, and then we can see that the MAEs of CNN-DOA are all more than $20^{\circ }$, which means that the CNN-DOA fails. However, the CFCN-DOA has lower errors under the lower SNR conditions. Even when the SNR is 0 dB, the accuracy can still reach 17.5°, but the MUSIC cannot work at this time. As the SNR increases, the MAE of CFCN-DOA continues to decline, all lower than that of MUSIC, reaching 1.45° at 20 dB SNR. We can also see the details in space-frequency pseudo-spectra of CFCN-DOA and MUSIC at 0 dB SNR, which are plotted in Figure 4c,d, respectively. In the area of $360^{\circ }\times 0-200\;\mathrm{Hz}$, the space-frequency pseudo-spectrum of MUSIC is almost flat, while the bright spots of CFCN can be identified. These characteristics can be seen more clearly from the average spatial pseudo-spectra in the area, displayed in Figure 4b. The CFCN-DOA has a higher resolution than MUSIC at a lower frequency. Note that CNN-DOA fails for low-frequency band-limited sources, and the estimated DOAs always tend towards some other unrelated points.

(b) Full-frequency source cases: The true DOA is also set to $182.5^{\circ }$. Simulation evaluation results are exhibited in Figure 5. Compared with the low-frequency band-limited case, the mean OSPAs of these methods are remarkably improved, which can be seen from Figure 5a. At this condition, CNN-DOA can work and reaches the same accuracy, i.e., $2.5^{\circ }$, with the MUSIC beyond 6 dB SNR, while the off-grid CFCN-DOA can further obtain higher accuracy. We can also see the superiority of CFCN-DOA from normalized space-frequency pseudo-spectra shown in Figure 5b,c. The spatial directivity of CFCN-DOA at each sub-band is more consistent than MUSIC. The spatial directivities of average spatial pseudo-spectra of MUSIC, CFCN-DOA, and CNN-DOA are enhanced in turn, which is displayed in Figure 5d. Normalizing the spatial spectrum for each sub-band, we can obtain a three-dimensional pseudo-beam pattern of CFCN, which is shown in Figure 5e. Then, the −3 dB pseudo-beam width in the frequency range 50–1500 Hz can be calculated by statistics at 20 dB SNR (See Figure 5f). The pseudo-beam width of CFCN-DOA is lower than the MUSIC at each sub-band, especially at 50 Hz CFCN-DOA reaching a pseudo-beam of less than $90^{\circ }$ MUSIC has nearly $160^{\circ }$ width. Since testing signals are similar to the training data set, the CNN-DOA performs very well, and the accuracy reaches the limit, i.e., the grid-gap, until the SNR is 20 dB. At this moment, the off-grid CFCN-DOA can achieve $1.45^{\circ }$.

(ii) Dual-source situation: The true DOAs of two sources are set to $92.5^{\circ }$ and $227.5^{\circ }$, respectively. Simulation results are counted in Figure 6. From Figure 6a–c, we can conclude that CNN-DOA cannot estimate multiple sources whether the frequency ranges are overlapping or non-overlapping, or the band is limited or non-limited. Although the other two methods can work in these three situations, MUSIC fails when the SNR is less than 20 dB under overlapping low-frequency, 6 dB under non-overlapping frequency, and 10 dB under overlapping full-frequency. On the contrary, CFCN-DOA is more capable of fighting low-SNR situations. The accuracy under the three conditions mentioned is $9.18^{\circ }$, $7.39^{\circ }$, and $10.48^{\circ }$, respectively. The space-frequency pseudo-spectra of CFCN under the three conditions mentioned are shown in Figure 6d–f respectively, and those of MUSIC are plotted in Figure 6g–i, respectively. From the space-frequency pseudo-spectra in the critical conditions of these MUSIC failures, CFCN-DOA can highlight the more spatial features of different frequencies. This also further verifies the generalization ability of the CFCN-DOA method based on learning space-frequency pseudo-spectrum.

## 5. Experimental Verification in Semi-Anechoic Chamber

**Figure 7:**
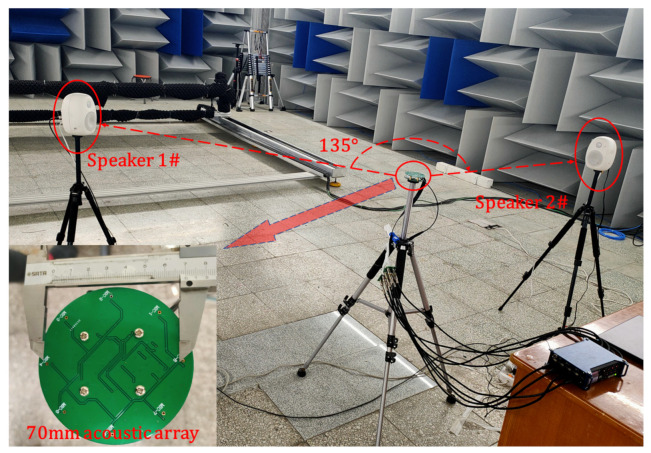
The settings of the semi-anechoic chamber experiment.

To verify the actual generalization performance of the proposed algorithm, we implement the semi-anechoic room test. The indoor noise is less than 30 dB. The experimental scene layout is shown in Figure 7. We adopt Hivi speakers as acoustic sources and use the recorder Zoom-F8n as the acquisition equipment. The distance between the speaker and the acoustic array is 1.4 m. The angular interval of the dual sources is set to 135°. Single/dual-source tests in different frequency ranges are carried out. Then, the simulation data are replayed. The experiment time of each test is more than 40 s, and the data are reprocessed with a 512-point Hanning window and 50% overlapping. Then, the first 200 frames of data are selected for statistics. Considering the limitation of the front-end MEMS microphone frequency response parameters, the processing frequency range is set to 50–1500 Hz.

**Figure 8:**
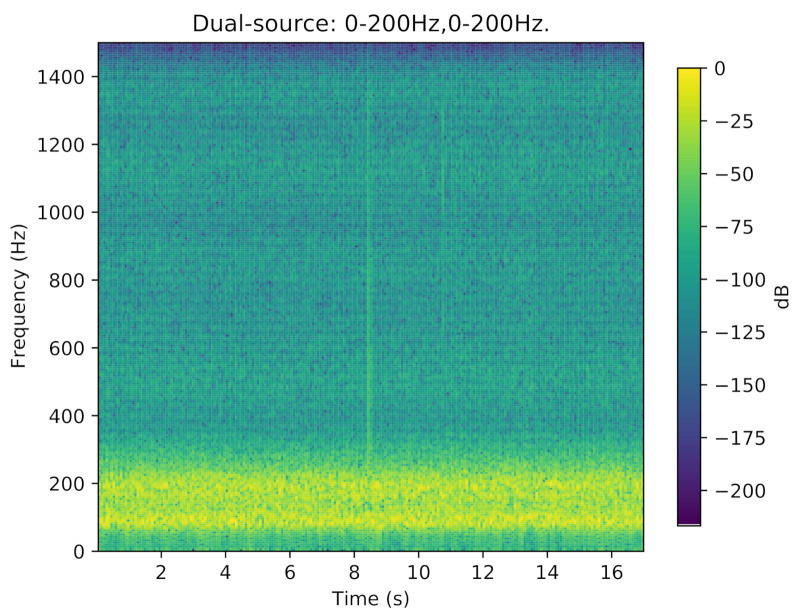
The frequency spectrum of a sensor receiving a signal from harmonic interference under the dual-source 0–200 Hz condition.

The results of the experiment in a semi-anechoic chamber are summarized as follows. Table 3 describes the mean OSPAs under all the conditions. Due to the limitation of the speaker frequency response, the speakers can not play pure low-frequency signals, and their harmonic components usually pollute the high-frequency parts. For instance, Figure 8 shows the frequency spectrum of a sensor receiving signal from harmonic interference under the dual-source 0–200 Hz condition.

**Figure 9:**
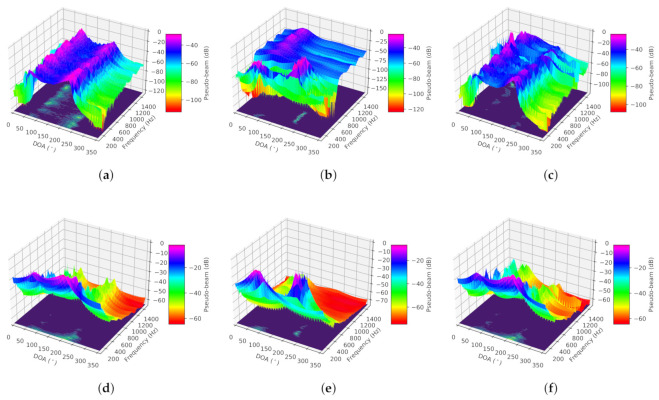
Space-frequency pseudo-spectra obtained by CFCN-DOA and CNN-DOA in the experiment in the semi-anechoic chamber. (**a**) CFCN under the overlapping low-frequency dual-source condition. (**b**) CFCN under the non-overlapping frequency dual-source condition. (**c**) CFCN under the overlapping full-frequency dual-source condition. (**d**) MUSIC under the overlapping low-frequency dual-source condition. (**e**) MUSIC under the non-overlapping frequency dual-source condition. (**f**) MUSIC under the overlapping full-frequency dual-source condition.

Coupled with the quiet environment in the semi-cancellation room, CNN-DOA can give full play to its performance. For the single-source low-frequency case, CNN-DOA can reach $3.42^{\circ }$ in such a quiet environment. However, CNN-DOA cannot deal with multi-source cases. The proposed off-grid methods have higher accuracy than MUSIC. If the frequency ranges of sources are non-overlapping, the accuracy of CFCN-DOA is up to $1.14^{\circ }$. Note that the error of CFCN-DOA under the dual-source overlapping full-frequency condition is larger than that under the dual-source overlapping low-frequency condition because the improved estimation performance caused by contaminated high-frequency signals for the low-frequency case is greater than the effect of frequency overlap for the full-frequency case. From Figure 9, the generalization performance is further verified by the high directivity at the space-frequency area. Note that the space-frequency pseudo-spectrum of the CFCN in Figure 9a spreads to the high-frequency range due to the weakly leaking high-frequency harmonic signals from speakers.

## 6. Discussion

From the simulation and semi-anechoic chamber experiments, the traditional CNN-based models have weak generalization ability for band-limited signals or multi-source, while the CFCN-based off-grid method can overcome these difficulties and its resolution and accuracy are better than MUSIC. There are two critical differences between these two deep learning-based methods.

(i) On the one hand, the input data of the two methods are the same, but their labels are different, i.e., the spatial pseudo-spectra as the labels of CNN-based methods and space-frequency pseudo-spectra as the labels of CFCN-based methods. The spatial features of each sub-band can be learned individually by the backbone of CFCN. In this way, other sources with limited frequency bands that do not match the features in the training data set can still be located.

(ii) On the other hand, the backbones of these two methods are different. The architecture of FCN can be split into an independent network for each sub-band, and the structure of each network is consistent. Then, CFCN can have the same interpretation capability on each sub-band. On the contrary, the features of all sub-bands are fused and mapped to the spatial pseudo-spectrum by the fully connected network in CNN-based methods. In this way, the model is more sensitive to frequency features.

Compared with traditional MUSIC-based methods, CFCN-based approaches richer nonlinear interpretation capabilities. The higher resolution and accuracy can be achieved by adjusting the network structures and related regressors. Although the CFCN has slightly high computational requirements, it is quite feasible to achieve online real-time processing for the current computing power.

## 7. Conclusions

In this paper, we propose an off-grid deep learning-based DOA estimation algorithm, which is based on the circularly fully convolutional network (CFCN). The backbone of this network is trained by the data set labeled by space-frequency pseudo-spectra and provides on-grid DOA proposals. Then, the regressor is developed to estimate the precise DOAs in the corresponding grid proposals. The simulation and semi-anechoic chamber experiment results show that under the conditions of single/dual sources with different band-limited/SNRs, the proposed algorithm is superior to existing methods in terms of generalization ability, resolution, and accuracy. Especially for the case of dual-source low-frequency and 12 dB SNR, CFCN can still distinguish multiple sources with an accuracy of $8.26^{\circ }$, while MUSIC and CNN-DOA fail at this time. Also, the −3 dB pseudo-beam width of CFCN reaches $90^{\circ }$ at 50Hz, which is much lower than the $160^{\circ }$ width of MUSIC. In future work, we hope that this proposed method can be extended to the case of coherent signals.

## Figures and Tables

**Table 1:** Comparison of different model parameters, accuracy, and total floating point operations (FLOPs).

The Structure of the Model	Number of Parameters (in Millions)	Accuracy (%)	Total FLOPs (Million)
CNN + FC [24]: K2-[64] × 7-FC[[512] × 2, 72]	8.48	89.4	53.67
CFCN: L4-K4-[[128] × 3, 72]	0.1689	92.74	345.66
CFCN: L4-K4-[72] × 4	0.0628	91.2	128.83
CFCN: L4-K2-[[128] × 3, 72]	0.0847	90.93	173.84
CFCN: L5-K4-[[128 × 4], 72]	0.2346	95.1	479.88
CFCN: L5-K2-[72] × 5	0.0420	94.5	86.53
CFCN: L5-K4-[72] × 5	0.0836	92.1	171.43
CFCN: L6-K4-[[128] × 5, 72]	0.3569	1.2	614.09
CFCN: L6-K4-[72] × 6	0.1044	98.5	214.02
CFCN: L6-K2-[72] × 6	0.0524	89.2	107.98
CFCN: L7-K4-[72] × 7	0.1252	1.3	256.62
CFCN: L7-K2-[72] × 7	0.0629	92.8	129.42
CFCN: L8-K2-[72] × 8	0.0733	95.5	150.86
CFCN: L9-K2-[72] × 9	0.0837	96.7	172.31
CFCN: L10-K2-[72] × 10	0.0942	97.1	193.75
CFCN: L11-K2-[72] × 11	0.1046	97.8	215.2
CFCN: L12-K2-[72] × 12	0.1151	1.4	236.64

**Table 2:** Comparison of different model average computing time.

	CNN + FC [24]: K2-[64] × 7-FC[[512] × 2, 72]	MUSIC	CFCN: L6-K4-[72] × 6 + Regressor
CPU	6.6 ms	486 ms	9.7 ms
GPU	1.2 ms	-	2.2 ms

**Table 3:** Mean OSPAs for the experiment in semi-anechoic chamber (Unit: ${}^{\circ }$).

Methods	Single-Source, Low-Frequency, (SNR = 81.5 dB)	Single-Source, Full-Frequency, (SNR = 89.9 dB)	Dual-Source, Overlapping Low-Frequency, (SNR = 120 dB)	Dual-Source, Non-Overlapping Low-Frequency, (SNR = 120 dB)	Dual-Source, Overlapping Full-Frequency, (SNR = 116 dB)
CFCN-DOA	2.15	0.15	1.52	1.14	3.45
MUSIC	4.64	2.76	6.84	2.56	3.75
CNN-DOA	3.42	1.23	28.89	33.04	26.75

## Data Availability

All the simulation/experimental data and codes will be made available on request to the correspondent author’s email with appropriate justification.
